# Protective effect of hydrogen sulfide on renal injury in the experimental unilateral ureteral obstruction

**DOI:** 10.1590/S1677-5538.IBJU.2014.0090

**Published:** 2015

**Authors:** Murat Dursun, Alper Otunctemur, Emin Ozbek, Suleyman Sahin, Huseyin Besiroglu, Ozgur Doga Ozsoy, Mustafa Cekmen, Adnan Somay, Nurver Ozbay

**Affiliations:** 1 Department of Urology, Bahcelievler State Hospital, Istanbul, Turkey; 2 Department of Urology, Okmeydani Training and Research Hospital, Istanbul, Turkey; 3 Department of Urology, Katip Celebi University, Ataturk Training and Research Hospital, Izmir, Turkey; 4 Department of Biochemistry, Kocaeli University, Kocaeli, Turkey; 5 Department of Pathology, Fatih Sultan Mehmet Training and Research Hospital, Istanbul, Turkey

**Keywords:** Ureteral Obstruction, Hydrogen Sulfide, Nephrogenic Fibrosing Dermopathy, Oxidative Stress

## Abstract

**Introduction/Objective:**

Ureteral obstruction is a common pathology and causes kidney fibrosis and dysfunction at late period. In this present study, we investigated the antifibrotic and antiinflammatory effects of hydrogen sulfide on kidney damage after unilateral ureteral obstruction (UUO) in rats.

**Materials and Methods:**

24 rats were divided into four groups. Group 1 was control, group 2 was sham, group 3 included rats with UUO and group 4 rats with UUO which were given sodium hydrogen sulfide (NaHS)-exogenous donor of hydrogen sulfide (intraperitoneally 56μmoL/kg/day). After 14 days, rats were killed and their kidneys were taken and blood analysis was performed. Tubular necrosis, mononuclear cell infiltration and interstitial fibrosis were determined histopathologically in a part of the kidneys; nitric oxide (NO), malondialdehyde (MDA) and reduced glutathione (GSH) levels were determined in the other part of the kidneys. Urea-creatinine levels were investigated by blood analysis. Statistical analyses were made by the Chi-square test and one-way analysis of variance (ANOVA).

**Results:**

There was no significantly difference for urea-creatinine levels among groups. Pathologically, there was serious tubular necrosis and fibrosis in group 3 and there was significantly decreasing of tubular necrosis and fibrosis in group 4 (p<0.005). Also, there was significantly increase of NO and MDA levels and decrease of GSH levels in group 3 compared to other groups (p<0.005).

**Conclusions:**

hydrogen sulfide prevents kidney damage with antioxidant and antiinflammatory effect.

## INTRODUCTION

Obstructive nephropathy is a common cause of renal insufficiency in children and adults. Decreases in renal blood flow and glomerular filtration occur after obstruction. Increased hydrostatic pressure causes damage to the tubule-interstitial compartment of the kidney (1). Apoptosis in tubular cells, capillary rarefaction, and interstitial cell inflammatory infiltration can be observed. The ensuing progressive fibrosis results in loss of parenchyma (2, 3). The obstruction can occur at any level of the urinary tract. The most common cause of obstruction in adults is urolithiasis, while obstructive nephropathy in children is mostly congenital (4).

Unilateral ureteral obstruction (UUO) is a well-established model known to imitate the process of obstructive nephropathy in a simple, accelerated and species-independent manner (5). In recent years, recovery of renal morphology following the relief of unilateral ureteral obstruction (UUO) has been examined in neonatal rats. Interestingly, it has been demonstrated that progressive tubule-interstitial and glomerular damage persisted in the obstructed and contralateral kidney and a decrease in glomerular filtration rate (GFR), and an increase in proteinuria occurred at the end of 1 year after relief of UUO (5, 6). Reactive oxygen species (ROS) are a recently recognized mechanism in the pathogenesis of UUO in experimental studies (7). Increased lipid peroxidation has been reported in renal cortexes of UUO animals. It has been shown that oxidative stress in UUO contributes to the development of tubulo-interstitial lesions and renal fibrosis. Various factors with complex cellular and molecular interactions have also been proposed as possible causes that lead to tubulo-interstitial lesions and renal fibrosis (8). Consequently, new therapy approaches are needed to prevent progression of renal injury along with surgical intervention. Therefore, concomitant treatment with an antifibrotic agent at the time of relief of UUO may prevent deterioration of renal function due to fibrosis. As previously reported, one of these agents may be hidrogen sulfide (H_2_S). For decades, hydrogen sulfide (H_2_S) has been known as a toxic gas, and, together with nitric oxide (NO) and carbon monoxide (CO), it is currently recognized as an endogenous gaseous physiological molecule (9). H_2_S is synthesized from cysteine by two pyridoxal-5′-phosphate dependent enzymes, cystathionine β-synthase (CBS) and cystathionine γ-lyase (CSE), and a pyridoxal-5′-phosphate-independent enzyme, 3-mercaptpyruvate sulfurtransferase (3-MST), in most mammalian tissues, including the kidney (10, 11). Progression of fibrosis is associated with oxidative stress, inflammatory response, vascular tone, and intracellular signaling pathways. Recent studies in human and animal have demonstrated involvement of H_2_S in those factors in various diseases, including atherosclerosis, ischemia and reperfusion (I/R) injury, hypertension, and end-stage renal disease (ESRD) (10, 11). In a previous study, H_2_S supplementation was associated with the suppressions of oxidative stress, inflammation and nitrosative stress (12).

Because of these effects of H_2_S, in this study we investigated the role of H_2_S in renal damage due to UUO. We used an exogenous donor of hidrogen sulfide-sodium hidrogen sulfide. We evaluated the antifibrotic, antinflammatory and antioxidative effects of H_2_S in rat kidneys.

## MATERIALS AND METHODS

### 

#### Drugs and Animals

Male Wistar albino rats (200–250 g) were housed in clean plastic cages in a temperature and humidity-controlled facility with a constant 12 h light/dark cycle with free access to food and water. The use of animals and the experimental protocol were approved by the Institutional Animal Care and Use Committee and animals were treated in accordance with the Guide for the Care and Use of Laboratory Animals of Research Council. Like previous study, sodium hydrogen sulfide (NaHS)-exogenous donor of H_2_S (Merck, Schuchardt, OHG, 85662 HOHENBRUNN, Germany), was administered intraperitoneally 56μmoL/kg/day for 14 days (13).

#### Experimental design

One week after acclimatization, UUO was induced. Briefly after induction of general anesthesia by intraperitoneal injection of thiopental (100mg/kg), the abdominal cavity was exposed via midline incision and the left ureter was ligated at 2 points with 4–0 silk. The sham-operated rats had their ureters manipulated but not ligated. All rats were given amikacin sulfate (6mg/kg, intramuscularly route) before operation (14).

After a quarantine period of 7 days, 24 rats were randomly divided into four groups, each consisting of six animals as follows: Rats in group 1 were control; Rats in group 2 were submitted to sham operation; Rats in group 3 underwent unilateral ureteral ligation and received no treatment; Rats in group 4 were subjected to unilateral ureteral ligation and received NaHS (intraperitoneally 56μmoL/kg/day) for 14 days. At this time, no animals showed symptoms of pyonephrosis and no one died because of pyonephrosis. So, we did not have to replace any animals. After 15 days, rats were killed and their kidneys were taken and blood analysis was performed. Tubular necrosis, mononuclear cell infiltration and interstitial fibrosis scoring were determined histopathologically in a part of kidneys; nitric oxide (NO), malondialdehyde (MDA) and reduced glutathione (GSH) levels were determined in the other part of the kidneys. Urea and creatinine levels were investigated by blood analysis.

#### Biochemical Assays

Twenty four hours after the administration of the last doses of NaHS, on 15th day, rats were anesthetized by intraperitoneal injection of ketamine and sacrificed. Renal cortical tissues were separated into two parts for biochemical analysis and light microscopic examination. Blood samples were also taken by cardiac puncture to assess the serum levels of urea and creatinine concentrations. In frozen tissues biochemically malondialdehyde (MDA), end product of lipid peroxidation, reduced glutathion (GSH), nonenzymatic antioxidant, and total nitrite, a stable product of nitric oxide (NO), were evaluated as a means of oxidative stress. Renal impairment was assessed by serum urea and creatinine levels, as well as by the kidney histology. Serum urea and creatinine levels were determined with an autoanalyzer (Syncron LX20, Ireland) by using commercial Becman Coulter diagnostic kits. Kidney tissue (300mg) was homogenized in icecold tamponade containing 150mM KCL for determination of MDA. MDA levels were assayed for products of lipid peroxidation. MDA referred to as thiobarbituric acid reactive substance, was measured with thiobarbituric acid at 532nm using a spectrofluorometer, as described previously. GSH was determined by the spectrophotometric method, which was based on the use of Ellman's reagent. Total nitrite (NOx) was quantified by the Griess reaction after incubating the supernatant with Escherichia coli nitrate reductase to convert NO_3_ to NO_2_. Griess reagent (1mL 1% sulfanilamide, 0.1% naphtyl-ethylenediamine hydrochloride, and 2.5% phosphoric acid; Sigma Chemical Co., St. Louis, MO, USA) was then added to 1mL of supernatant. The absorbance was read at 545 nm after a 30-min incubation. The absorbance was compared with the standard graph of NaNO_2_, obtained from the reduction of NaNO_3_ (1–100mmoL/L). The accuracy of the assay was checked in two ways; the inter- and intraassay coefficients of variation were 7.52 and 4.61%, respectively. To check conversion of nitrate to nitrite (recovery rate), known amounts of nitrate were added to control plasma samples; these samples were deproteinized and reduced as above.

#### Histopathological Examinations

Histopathological evaluation of the kidney tissues was done. Paraffin embedded specimens were cut into 6μm thickness and stained with Hematoxylin-Eosin stain for light microscopic examination using a conventional protocol (Olympus, BH-2, Tokyo, Japan). A semi-quantitative evaluation of renal tissues was accomplished by scoring the degree of severity according to previously published criteria (15). All sections of kidney samples were examined for tubular necrosis. Briefly, a minimum of 50 proximal tubules associated with 50 glomeruli were examined for each slide and an average score was obtained. Severity of lesion was graded from 0 to 3 according to the percentage of tubular involvement. Slides were examined and assigned for severity of changes using scores on scale in which (0) denotes no change; grade (1) changes affecting <25% tubular damage (mild); grade (2) changes affecting 25–50% of tubules (moderate); grade (3) changes affecting >50% of tubules (severe). Histopathological evaluation was performed on left kidney tissues. Paraffin-embedded specimens were cut into 5mm thick sections and stained with Hematoxylin & Eosin and Masson's trichrome for examination under the light microscope (BH-2; Olympus, Tokyo, Japan). To evaluate leukocyte infiltration, the widening of interstitial spaces with focal leukocyte infiltration was assessed in five randomly chosen sections prepared from each kidney sample. For each section, the average number of leukocytes per 0.28mm^2^ was calculated from these leukocyte-infiltrated foci using a high-power microscopic field (X400). To estimate the grade of interstitial fibrosis, the interstitial area that was stained green with Masson's trichrome was evaluated as a percentage of the total examined area in five randomly chosen sections prepared from each kidney sample using an image analyzer (Leica; Leica Micros Imaging Solutions, Cambridge, UK). For each section, interstitial space widening with focal leukocyte infiltration and interstitial fibrosis was assessed in high-power fields (X400) to quantify the results. The Banff classification of kidney pathology was used for scoring the degree of mononuclear cell infiltration and interstitial fibrosis. The score was graded from 0 to 3, depending on the severity of histological characteristics (16).

### Statistical analysis

Results of all groups are shown as mean values ± standard deviation (SD). Statistical analyses of the histopathologic evaluation of the groups were carried out by the Chi-square test and biochemical data were analyzed by the one-way analysis of variance (ANOVA). The significance between two groups was determined by the Dunnett's multiple comparison test, and P<0.05 was accepted as statistically significant value.

## RESULTS

### 

#### Biochemical Variables in Plasma and Tissue

There was no significantly difference for urea-creatinine levels between groups (Table-1). Tissue MDA levels significantly increased in group 3 compared with groups 1, 2, and 4 (p<0.05). Rats with NaHS administration (group 4) showed reduced levels of lipid peroxidation as measured by MDA levels (Table-2). UUO also induced a significant increase in the tissue NO levels that have been prevented by NaHS (Table-2). The unilateral ureteral ligation was accompanied by a marked reduction in GSH level in the kidney tissues of rats (p<0.05), and treatment with NaHS partially elevated the GSH levels (Table-2).

**Table 1:** Effects of UUO alone and its combination with NaHS on plasma urea, creatinine levels in rats.

Parameters	Control (Group 1)	Sham (Grup 2)	UO (Grup 3)	UO+NaHS (Grup 4)
Urea (mg/dL)	29±7.4	28.7±8.1	30.2±9.5	29.9±10.3
Creatinine (mg/dL)	0.38±0.3	0.36±0.1	0.42±0.2	0.40±0.1

Values are expressed as mean ± SD for six rats in each group.

**Table 2:** - Effect of H_2_S on the Levels of Malondialdehyde, Glutathione and Nitric Oxide in Each Rat Group.

Parameters	Control (Grup 1)	Sham (Grup 2)	UO (Grup 3)	UO+NaHS (Grup 4)
NO (nmoL/g wet tissue)	24.83±3.95	24.63±3.28	38.90±6.82^a^	27.03±3.61^b^
MDA (nmoL/g wet tissue)	2.75±0.19	2.84±0.29	4.17±0.79^c^	3.07±0.45^d^
GSH (mg/g wet tissue)	2.25±0.10	2.25±0.04	1.20±0.73^e^	2.06±0.15^f^

Values are expressed as mean ± SD for six rats in each group

a, c, e significantly difference from control group (p=0.02, p=0.005, p<0.001).

b, d, f significantly difference from UO group (p=0.006, p=0.015, p<0.001).

**NO =** nitric oxide, **MDA =** malondialdehyde, **GSH =** reduced glutathione

#### Histopathologic Examinations Results

**Figure 1:**
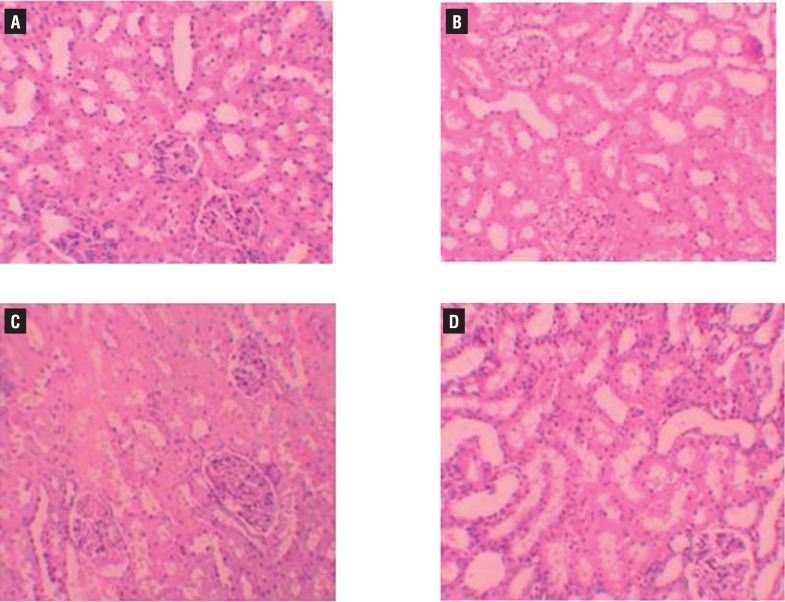
A) normal tubulus and glomerules in kidney kortex H&Ex200 (control group). B) normal tubulus and glomerules in kidney kortex H&Ex200 (sham group). C) severe tubules total necrosis, tutbular degeneration and epithelial vacuolization in proximal tubules H&Ex200 (UUO group). D) mild epithelial vacuolization in the proximal tubules and normal glomerules H&Ex200 (UUO+NaHS treated group).

**Figure 2:**
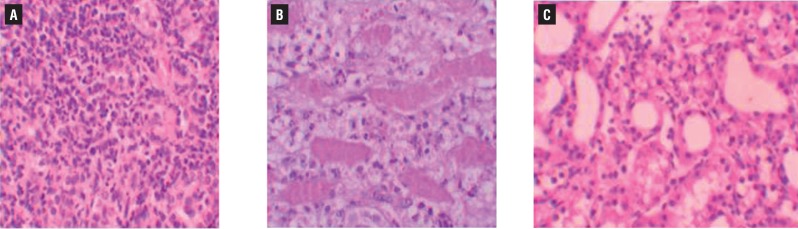
A) severe mononuklear leukocyte infiltration in the cortex of UUO group (hematoxylin & eosin x400) B) leukocyte infiltration was observed in the peritubular interstitium of the UUO (hematoxylin & eosin x400); c) leukocyte infiltration was reduced in the NaHS-treated group (hematoxylin & eosin x400).

**Figure 3:**
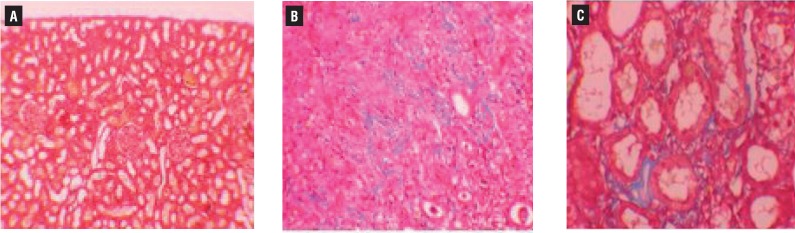
A) normal kidney morphology in a sham group, group (masson & trichrome x200). b) severe fibrosis was observed in the peritubular interstitium of the UUO, group (masson & trichrome x400). c) mild fibrosis was reduced in the NaHS-treated group (masson & trichrome x400).

Histopathologic examination of kidney showed no pathologic findings in control group (Figure-1a). In rats with UUO, there were mild and severe tubular necrosis in the proximal tubules compared with control and sham groups (Figures 1b and 1c). In rats treated with UUO+NaHS, despite the presence of mild tubular degeneration and tubular necrosis, the findings were less severe, and glomeruli maintained a better morphology when compared with UUO group (Figure-1d). Histopathologic examination was normal in rats with only sham operation (group 2) and in rats with no operation (group 1). Severe leukocyte infiltration was observed in the periglomerular and peritubular interstitium of the kidneys of the rats in group 3 with UUO (Figures 2a and 2b). Quantitative analysis of the focal leukocyte infiltration area in the interstitium showed that leukocyte infiltration was significantly reduced in rats that received UUO+NaHS (group 4) (Figure-2c). UUO caused a significant interstitial fibrosis in rats that received no treatment (group 3), and the percentage area of interstitial fibrosis in the kidney of rats with UUO that received no treatment was significantly greater than that of rats with UUO that received NaHS (group 4) (Figures 3a, 3b and 3c). These changes are summarized in Table-3.

**Table 3:** Semiquantitative analysis of tubular necrosis, interstitial fibrosis, mononuclear cell infiltration in control, sham, UO, and UO+H_2_S treated rats.

	Tubular necrosis					Interstitial fibrosis				Mononuclear cell infiltration			
	n	0	1	2	3	0	1	2	3	0	1	2	3
Control	6	6	0	0	0	6	0	0	0	5	1	0	0
Sham	6	6	0	0	0	6	0	0	0	5	1	0	0
UO^a^	6	0	0	4	2	0	1	3	2	0	1	3	2
UO+NaHS^b^	6	1	3	2	0	2	3	1	0	1	1	4	0

Score 0: no degeneration, 1: mild degeneration, 2: moderate degeneration, and 3: severe degeneration

a Statistical significant difference from the Sham group

b Statistical significant difference from the UO group and P < 0.05.

## DISCUSSION

Obstructive uropathy, caused by prevention of urine flow, results in permanent renal damage and loss of renal function. The obstruction can occur at any level of the urinary tract. The most common cause of obstruction in adults is urolithiasis, while obstructive nephropathy in children is mostly congenital (4). Acute obstruction of the ureter rapidly triggers a cascade of events in the kidneys. First, renal blood flow and the glomerular filtration rate drop. Within a few days, hydronephrosis starts to develop, followed by interstitial inflammatory infiltration, apoptosis, and necrosis.

The pathogenesis of renal fibrosis caused by UUO involves infiltration of the kidney by inflammatory cells including monocytes, activation and possible transformation of intrinsic renal cells, and interactions between infiltrating and resident cells. Reactive oxygen species (ROS) are a recently recognized mechanism in the pathogenesis of UUO in experimental studies (17). So we decided to measure the MDA, GSH, and nitric oxide (NO) content, as a means of oxidative stress. In our study confirmed through a quantitative survey the protective role of H_2_S on renal tissue damage after the induction of UUO in rats. Our results showed that the obstructed kidney had significantly higher tissue MDA, NO levels, and lower GSH levels along with more fibrosis. Our findings corroborate those of earlier studies demonstrating that an enhanced endogenous oxidative stress has a major role in the severity of UUO-induced acute renal failure (18, 19). On the other hand, H_2_S reduced the severity of injury, depressed the concentration of these cytokines and increased the antioxidative capacity.

Endogenous H_2_S has been proposed as a novel cytoprotective mediator (20), and there is growing evidence of direct and indirect antioxidant effects of H_2_S. In cell culture experiments, H_2_S/HS^−^ generated from NaHS has been shown to ‘scavenge’ detrimental pro-inflammatory oxidants, such as H_2_O_2_ (21), ClO^−^ (22), superoxide, ONOO^−^ and NO, inhibit cell death induced by these mediators as well as prevent oxidative modification of intracellular proteins (22) and LDL (low-density lipoprotein) (23). In neuronal cells, NaHS inhibited cell death induced by β-amyloid, mediated at least in part via antioxidant effects (24) and up-regulating intracellular glutathione synthesis through increasing cysteine uptake and elevating γ-glutamylcysteine synthetase activity. NaHS is also reported to degrade lipid peroxides (24), inhibit the expression and activity of NADPH oxidase and up-regulate thioredoxin-1 expression in vascular endothelial cells (25). Increased hepatic GSH synthesis and decreased lipid peroxidation are also observed with NaHS treatment in a murine hepatic ischaemia/reperfusion injury model (26).

Increased lipid peroxidation (LPO) has been reported in renal cortexes by the induction of excessive ROS in renal ischemic reperfusion (27). MDA is the product in the LPO process and is widely used as a reliable marker of tissue damage. In the present study, we found increased MDA levels in UUO group and as protective effect of H_2_S lower MDA levels in group determined by UUO+NaHS. The GSH antioxidant system is considered the most notable cellular protective mechanism. GSH has a very important role in protecting against oxygen free radical damage by providing reducing equivalents for several enzymes, as well as scavenging hydroxyl radicals and singlet oxygen. Its depletion is a common consequence of increased formation of ROS like UUO- induced nephrotoxicity. In group given UUO+NaHS, we found increased GSH levels. However, our study have shown that H_2_S effects NO levels protectively similar to some previous studies with different antioxidant agents (28). H_2_S can inhibit NO production and NF-kappaB activation in LPS-stimulated macrophages through a mechanism that involves the action of HO-1/CO (29). Because of that, in our study we found decreased NO levels in UUO+NaHS group compared to UUO group. These findings strongly indicate that H_2_S is important in protecting the kidney from UUO-induced injury through improvement in oxidant status.

In this study, the histopathologic examination of kidneys showed severe and extensive damage in UUO rats which have tubular necrosis and edema. This could be due to the formation of highly reactive radicals as a consequence of oxidative stress caused by UUO. The kidneys of the control group showed normal histological features, but the UUO group revealed more extensive and marked tubular necrosis. On the other hand, the tubules from rats of the UUO+NaHS group were nearly normal in histological appearance except for a slight desquamation and atrophy of the tubular epithelial cells. Similar changes were also reported by some studies who demonstrated structural changes in renal tissue of gentamicin-treated animals and its reversal by various agents (30).

In conclusion, the results reported here indicate that H_2_S exerts a preventive effect on UUO-induced kidney damage in rats by reducing oxidative stress. At present, hydronephrosis is an urological condition that needs a quick surgical treatment. But, surgery pre-operative phase might take longer than we planned. So, we can use NaHS treatment to prevent the kidney damage in clinics until the surgical treatment. One limitation in our study was that the underlying molecular mechanisms that are responsible for the positive effects of NaHS are yet to be determined. We therefore propose that NaHS supplementation therapy can be used for kidney protection in patients with UUO, such as with ureteral stones. Hovewer, further animal and clinical studies are needed to confirm our suggestion.
